# Problematic Social Media Use: Results from a Large-Scale Nationally Representative Adolescent Sample

**DOI:** 10.1371/journal.pone.0169839

**Published:** 2017-01-09

**Authors:** Fanni Bányai, Ágnes Zsila, Orsolya Király, Aniko Maraz, Zsuzsanna Elekes, Mark D. Griffiths, Cecilie Schou Andreassen, Zsolt Demetrovics

**Affiliations:** 1 Institute of Psychology, Eötvös Loránd University, Budapest, Hungary; 2 Doctoral School of Psychology, Eötvös Loránd University, Budapest, Hungary; 3 Institute of Sociology and Social Policy, Corvinus University of Budapest, Budapest, Hungary; 4 International Gaming Research Unit, Nottingham Trent University, Nottingham, United Kingdom; 5 Department of Clinical Psychology, University of Bergen, Bergen, Norway; Hospital Universitari de Bellvitge, SPAIN

## Abstract

Despite social media use being one of the most popular activities among adolescents, prevalence estimates among teenage samples of social media (problematic) use are lacking in the field. The present study surveyed a nationally representative Hungarian sample comprising 5,961 adolescents as part of the European School Survey Project on Alcohol and Other Drugs (ESPAD). Using the Bergen Social Media Addiction Scale (BSMAS) and based on latent profile analysis, 4.5% of the adolescents belonged to the at-risk group, and reported low self-esteem, high level of depression symptoms, and elevated social media use. Results also demonstrated that BSMAS has appropriate psychometric properties. It is concluded that adolescents at-risk of problematic social media use should be targeted by school-based prevention and intervention programs.

## Introduction

### Social media use

Social media use is currently one of the most popular leisure activities among adolescents (e.g., [1–3]). Social media (e.g., *Facebook*, *Instagram*, *Snapchat*, etc.) host virtual communities where users can create individual public and/or private profiles [4–6]. Users can access social media on different platforms (mobile or computer devices), for different activities (e.g., interacting with real-life friends, meeting others based on shared interest, chatting, mailing, sharing or creating pictures / videos, blogging, dating, playing games, gambling; [7–9]).

*Facebook* is one of the most popular social media among 13–17 years old adolescents in the USA [2]. According to a recent report, 71% of teenage social media users access more than one social media and 24% of adolescents are “almost constantly” online due to the widespread use and popularity of smartphones [2]. Furthermore, there is an increasing interest to explore and assess the characteristics and prevalence of problematic/excessive use of social media (e.g., [4, 10–14]).

### Problematic social media use

To date, there is no consensus among researchers regarding the definition of problematic social media use due to the conceptual confusion surrounding the classification of problematic internet use [15, 16]. Negative outcomes triggered by the excessive use of social media may have a detrimental effect on the personal, social, and/or professional lives of the users [8, 13, 17–20]. Lee, Cheung, and Thadani [21] argued that obsessive *Facebook* users had troubles in work, academic performance. and interpersonal relationships. For instance, Pantic and Damjanovic [22], Wegmann and Stodt [16], and Andreassen and Billieux [23] reported a significant positive correlation between depression symptoms and social media use, while Malik and Khan [24] found negative relationship between self-esteem and high levels of social media use.

Due to the lack of consistency in empirical studies, diagnosis of internet-related disorders has yet to be established based on the aforementioned theoretical constructs. Internet Use Disorder was suggested for consideration in the latest (fifth) edition of the Diagnostic and Statistical Manual of Mental Disorders (DSM-5; [25]). However, only one internet-related disorder—Internet Gaming Disorder—was included in Section 3 of the DSM-5. Another problem is that various synonyms of problematic social media use exist in the literature with different diagnostic suggestions including (among others) *Facebook* dependence [26], *Facebook* addiction [10], social networking addiction [27], *Twitter* addiction [28], social media addiction [11], and Social Media Disorder [29].

Different theoretical models provide explanations for the development of problematic social media use (e.g., cognitive-behavioral, social skill, or socio-cognitive models; [30]. These theoretical models have been developed from a clinical perspective, while the biopsychosocial model concerns behavioral addictions in general [14]. According to the biopsychosocial model [31], problematic social media use can be determined by a range of addiction symptoms including: mood modification (i.e., excessive social media use leading to specific changes in mood states), salience (i.e., total preoccupation with social media use), tolerance (i.e., increasing amounts of time using social media), withdrawal symptoms (i.e., negative feelings and psychological symptoms such as irritability, anxiety when social media use is restricted), conflict (i.e., interpersonal problems as a direct result of social media usage), and relapse (i.e., returning to excessive social media use after a period of abstinence).

### Assessing problematic social media use

To obtain a reliable prevalence rate of problematic social media usage, it is important to use psychometrically valid measurement tools. Due to the problem of inconsistencies regarding the definition of problematic, excessive, or addictive social media use, there is also a lack of reliable and valid psychometric scales to assess the phenomenon of problematic social media use. More specifically, the existing assessment tools are based on different diagnostic suggestions such as problematic internet use (e.g., Internet Addiction Test; [32–34], Internet Gaming Disorder [29]), or other aspects of addictive tendencies (e.g., withdrawal, loss of control, salience; [35, 36]. In addition, some of the measurement tools focus only on specific social media (e.g., *Facebook*; [14] such as the *Facebook* Addiction Symptoms Scale [37], the *Facebook* Addiction Scale [38], the Bergen *Facebook* Addiction Scale [10], and the *Facebook* Intrusion Questionnaire [39]).

Although the most recent data show that *Facebook* is the most popular and frequently used social media among adolescents [2], empirical research has shown that adolescents use more than one social media frequently (e.g., [2]). Therefore, the assessment tools are unable to follow the ever-changing trends in the area of social media use. Considering the increased usage of various social media among adolescents [1–3, 5] the questionnaires should assess all available social media and the total range of activities on these social media instead of one specific social media such as *Facebook* [14].

### Prevalence of problematic social media use

It is difficult to estimate the prevalence of problematic social media use due to the use of various assessment tools and the lack of a consensual definition of problematic social media use. Furthermore, recent research has demonstrated that problematic social media use has a higher prevalence among female users than males [11, 13, 40, 41]. Unfortunately, in studies that have assessed different aspects of problematic social media use, the gender distribution was usually frequently imbalanced in that women were typically over-represented [11, 12, 15, 26, 29, 37, 42–44], and may be explained by the higher willingness of females to participate in such studies.

Due to different theoretical frameworks and psychometric assessments, the prevalence of problematic social media use might be underestimated or overestimated. Previous studies have reported different prevalence rates relating to problematic social media use. For instance, Olowu and Seri [44] reported a prevalence rate of 2.8% of addicted social media use among college students, while Jafarkarimi and Sim [43] reported a prevalence rate of 47% being addicted to *Facebook* among a sample of college students. Explanations for the large difference in problematic social media use prevalence rates might be the non-representative (self-selected and typically small participant) samples and different cultural groups examined (e.g., Chinese, Australian, Nigerian college students, Dutch adolescents [12, 15, 26, 29, 37, 42–44]. Moreover, to date, there has only been one nationwide survey assessing problematic (ie., addictive) social media use [11] that examined the associations between problematic social media use, narcissism, and self-esteem, and between problematic use of social media, attention-deficit/hyperactivity, obsessive-compulsiveness, anxiety, and depression on cross-sectional convenience sample of 23,532 Norwegians (although the sample was not nationally representative).

Furthermore, no studies have examined the prevalence of problematic social media use utilizing a representative adolescent sample. Furthermore, only a few studies exist concerning problematic social media use among adolescents (e.g., [29]). Previous studies have reported an increased popularity of social media use among adolescents [1–4, 6] and the increased number of adolescent social media users could explain the higher prevalence of problematic usage in this group [4, 6]. Consequently, the aim of the present study was twofold:

1. To test the psychometric properties of the Bergen Social Media Addiction Scale (BSMAS) using a nationally representative (Hungarian) adolescent sample.
2. To assess the prevalence of problematic social media use in a nationally representative adolescent sample.

## Methods

### Participants and procedure

The data were collected in March 2015 as part of the European School Survey Project on Alcohol and Other Drugs (ESPAD; [45]) that included a nationally representative adolescent sample. The target population was adolescents aged 16 years. In 2015, Hungary included a short section to assess internet and social media use in addition to the original questionnaire developed by the ESPAD Committee.

To obtain a representative sample, two different grades (9^th^-10^th^) were included in the Hungarian data collection, each containing a proportion of the target population. To reduce sampling error, the grades were divided into non-overlapping, homogeneous subgroups. The variables to ensure the representativeness of the adolescent sample were as follows: region (central/western/eastern Hungary), grade (9th, 10th), and type of class (secondary general, secondary vocational, vocational classes). The data were collected anonymously from the students in the classrooms of the schools by research assistants.

The refusal rate was 7% on the level of the primary sampling unit (i.e., classes) that led to skewed nonresponse. To match the composition of the respondents with the sampling frame, data were weighted by strata with the matrix weighting method recommended by the Education Information System 2014/2015 (KIR-STAT; Elekes, 2015). The total sample consisted of 6,664 participants (50.94% male). The youngest participants were 15 years old, while the oldest were 22 years (mean age 16.62 years; SD = 0.96). The wide age range was due to a very small number of older students still attending the 9^th^ or 10^th^ grades at the age of 19 years or older at the time of data collection. The questions concerning internet use and social media use were included for this nationally representative sample of 9^th^-10^th^ graders in secondary general and secondary vocational schools. Participant data with severe incompleteness or inconsistencies were excluded (3.72% of the sample), in addition to those participants who did not use the internet and/or any social media (an additional 6.83% of the original sample). After removing these participants, the final sample size was 5,961 (89.45% of the total sample).

This study was approved by the Scientific Ethical Committee of Corvinus University of Budapest. The study design was based on an international protocol approved by the European School Survey Project on Alcohol and Other Drugs (ESPAD) Assembly, which was conducted in full compliance with the principles expressed in the Declaration of Helsinki. Written informed consent was requested from both the students and their parents (passive on behalf of their children).

### Measures

#### Socio-demographics questions

Information regarding gender, age, grade, and residence were collected.

#### Weekly social media use

To assess the adolescents’ weekly time spent on social media on computer or other devices (e.g., handled devices) two variables were combined: (i) ‘The last 7 days how many days did you use the internet for social networking?’ (Categories were ‘never’, ‘1 day’, ‘2 days’, …‘7 days’); (ii) ‘In the last 30 days on an average day how many hours did you use the internet for social networking?’ (Categories were ‘I don’t use’, ‘less than half an hour’, ‘1 hour’, ‘2–3 hours’, ‘4–5 hours’ and ‘more than 6 hours’).

#### Bergen Social Media Addiction Scale (BSMAS)

To assess problematic social media use, the Bergen Social Media Addiction Scale (BSMAS; [11]) was used. The 6-item scale was adapted from the previously validated Bergen *Facebook* Addiction Scale (BFAS; [10]). The original scale specifically assessed problematic *Facebook* use during the last year. The scale incorporated the theoretical framework of the addiction components of the biopsychosocial model [31]. The BFAS was developed by selecting the items with the highest possible factor loadings for each component (i.e., salience, mood modification, tolerance, withdrawal symptoms, conflict, and relapse) from an item-pool of 18 initial items. In the present study, the Bergen Social Media Addiction Scale (BSMAS) which is based on rephrasing of the BFAS, was to assess social media use in general over the past 12 months. The scale was translated to Hungarian and then back-translated by independent translators. The back-translation was then compared with the original scale and adjustments were made as necessary. The items are answered on a 5-point scale (“never” to “always”). The Cronbach’s alpha of the translated BSMAS was .85 in the present sample.

#### Rosenberg’s Self-Esteem Scale

Self-worth was assessed by the Hungarian version of the Rosenberg’s Self-Esteem Scale (RSES-HU; [46], Hungarian version, [47]). The RSES assessed global self-esteem (i.e., feeling of self-worth and self-acceptance) with 10 items on a 4-point scale (“strongly agree” to “strongly disagree”). The score range is between 10–40 and the higher the score, the higher the self-esteem. Cronbach’s alpha was 0.87 in the present sample.

#### Center of Epidemiological Studies Depression-Scale

Depressive mood was assessed with the 6-item short-form of the Center of Epidemiological Studies Depression-Scale (CES-D; [48]). The scale assesses the level of depressive symptoms but it was not designed to diagnose clinical depression. The instrument was translated and then back-translated by Hungarian experts in the addiction field. The back-translation was then compared to the original instrument and adjustments were made where necessary. The items of CES-D were answered on a 4-point scale (“rarely or never” to “most of the time”). The score range is 4–24, and a higher score indicates higher level of depressive symptoms. Cronbach’s alpha was 0.84 in the present sample.

### Statistical analysis

To test the one-factor model of the BSMAS, confirmatory factor analysis (CFA) was performed with maximum likelihood estimation with robust standard error (MLR) in Mplus 7.3 [49]. To evaluate the model fit, a *p*-value of Chi-square (χ^2^) higher than .05 was used for the test of close fit [50]. Additional fit indices were also included: the comparative fit index (CFI), the Tukey-Lewis Fit Index (TLI), the root mean square error of approximation (RMSEA) and its 95% confidence interval (90% CI), and standardized root mean square residual (SRMR). To indicate a good fit of the model, both CFI and TLI values have to be over than .90 or over .95 [51], while the values of RMSEA and SRMR should be less than .05 and .10 respectively [51–53].

In order to identify the groups of adolescents with high risk of problematic social media use, a mixture modeling technique called latent profile analysis (performed in Mplus 7.3) was used. Latent profile analysis is a mixture modeling technique to identify groups of people (categorical output variable of the analysis) according to their responses to certain continuous variables (in the present study’s case, the scores given on the six items of the BSMAS). Individuals with similar responses are classified in the same group [54]. Latent profile analysis was performed with 2 to 4 classes in the full sample (n = 5,961). To determine the number of latent classes, several indices were used, such as the measures of parsimony of each model (i.e., Akaike Information Criteria—AIC, Bayesian Information Criteria—BIC, and the Sample Size Adjusted Bayesian Information Criteria—SSABIC). The lower values on these indicators, the more parsimonious the model. The entropy criterion and the interpretability of clusters were also examined. In the final determination of the number of classes, the likelihood-ratio difference test (Lo-Mendell-Rubin Adjusted LRT Test) was used that statistically compares the fit of the estimated model with a model having one less class than the estimated one. The *p*-value of less than .05 suggests the tested model fits better than the model with one less class [49].

To test the construct validity of the BSMAS, the LPA classes were compared along a number of variables relevant to the phenomenon of SOCIAL MEDIA use (i.e., gender, SOCIAL MEDIA use hours/ week, level of self-esteem, level of depression). For these comparisons, Wald’s Chi-square test of mean equality for latent class predictors in mixture modeling was used because it takes into consideration the probabilistic nature of the LPA classes (for description of the analysis, see www.statmodel.com/download/meantest2.pdf).

To determine the optimal cut-off point for the BSMAS, a sensitivity analysis was performed and the group with the highest risk of problematic social media use (based on the results of the LPA analysis) was considered as the “gold standard”. The present authors are aware that this method does not replace the clinical validation process, however, the authors believe that this is better than using completely ad-hoc cut-off points as several other studies do. The sensitivity, specificity, positive and negative predictive value (PPV and NPV, respectively), and the accuracy of each cut-off threshold were calculated and compared to identify the cut-off value with the best indicators. Sensitivity is the proportion of true positive cases belonging to the at-risk group based on the LPA (the “gold standard” group in this case). Specificity is the proportion of the true negatives among those who do not belong to the at-risk group based on the LPA [55, 56]. PPV is the proportion of true positives among all participants who scored positive on the test. NPV was defined as the proportion of true negatives among all participants with negative test results [56, 57]. Finally, accuracy measures the proportions of true negatives and true positives among all participants [56, 57].

All analyses were conducted on the weighted sample. Missing data were treated with Full-information maximum likelihood (FIML) method [49]. Statistical analyses were carried out with Mplus 7.3 [49] and IBM SPSS Statistics for Windows, Version 22.0 [58].

## Results

### Descriptive statistics

The final sample only comprised those participants who reported using *the internet and social media* (n = 5,961, 89.45% of the total sample). Approximately half (49.17%) of the sample was male (n = 2931). Age ranged between 15 and 22 years (mean age 16.60; SD = 0.94). The mean number of hours using social media was 23.16 hours per week (SD = 15.57). There was a significant difference in weekly social media use between male and female adolescents (mean time_male_ = 20.53 hours, SD_male_ = 15.71; mean time_female_ = 25.71 hours, SD_female_ = 15.00; U = 3672101, *p*< 0.001; r = -0.17).

### Confirmatory factor analysis

A one-factor model with the six components (salience, tolerance, mood modification, relapse, withdrawal, conflict) as indicator variables was tested with confirmatory factor analysis. The analysis provided an acceptable fit to the data (χ^2^ = 5836.190 df = 15 *p*< 0.001; CFI = 0.950; TLI = 0.917; RMSEA = 0.073 (0.066–0.080) Cfit>0.90; SRMR = 0.034). All factor loadings were above the recommended threshold (>.50) and ranged from .598 to .814.

### Latent profile analysis

The latent profile analysis was performed on the six items of the BSMAS, and according to the criteria, the three-class solution was selected as the best-fitting model (see Table 1). The AIC, BIC, and SSABIC values decreased continuously as more classes were added to the analysis. However, the scale of decrease somewhat diminished after the third latent class was added. Based on the L-M-R test, the three-class solution was accepted. The entropy of the two-class solution was the highest, but the entropy of the three-class solution was also adequate.

**Table 1:** Results of the Latent Profile Analysis.

Fit indices for the Latent Profile Analysis (LPA) of the social media use									
Model	Log-likelihood	Replicated log-likelihood	Nr. of free parameters	AIC	BIC	SSABIC	Entropy	LMR-LRT test	*p*
2 classes	-43837	Yes	19	87711	87837	87778	0.96	12838	<0.0001
**3 classes**	**-42241**	**Yes**	**26**	**84534**	**84708**	**84626**	**0.94**	**3140**	**<0.05**
4 classes	-41097	Yes	33	82260	82481	82376	0.95	2251	0.69

*Note*: AIC = Akaike Information Criterion, BIC = Bayesian Information Criterion, SSABIC = sample size adjusted BIC, LMR-LRT = Lo–Mendell–Rubin Likelihood Ratio Test. Bold data indicate that the three-class solution was selected as a result of the LPA analysis.

**Fig 1:**
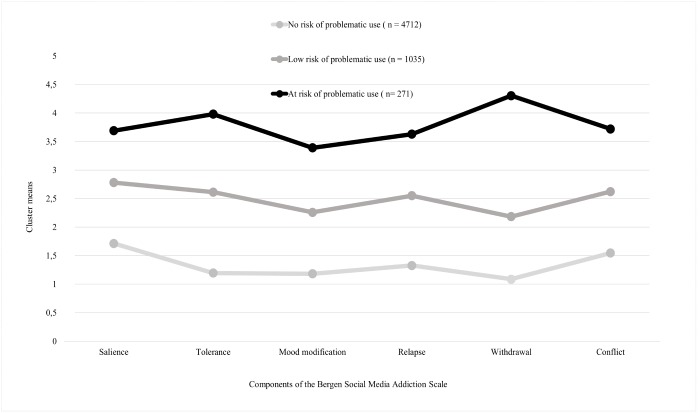
The Three Classes Obtained from the Latent Profile Analysis.

The features of the three classes are presented in Fig 1 and Table 2. The first class named ‘no-risk’ class represents the majority of social media users (78.3% of social media users; 70.7% of the total sample) who had the lowest scores on the BSMAS. The second class of social media users represents ‘low risk’ of problematic use (17.2% and 15.5% respectively), while the third class represents the population of ‘at-risk’ problematic social media users (4.5% and 4.1%, respectively). In the ‘at-risk’ group, ‘withdrawal’ and ‘tolerance’ criteria showed elevated levels compared to the other dimensions. Members of this class (i.e., those at-risk of problematic use) were likely to (a) be female, (b) use the internet and social media for more than 30 hours per week, (c) have lower self-esteem and higher level of depressive symptoms than social media users of the other two classes (Table 2).

**Table 2:** Comparison of the Three Latent Classes: Testing Equality for Latent Class Predictors.

	No risk class (n = 4712)	Low risk class (n = 1035)	At-risk class (n = 271)	Overall test	
				Wald χ^2^	*p* value
Gender (male %)	50.36_a_	44.51_b_	41.2_b_	13.58	0.001
Age (years); Mean (SE)	16.60 (0.02)_a_	16.61 (0.03)_a_	16.69 (0.06)_a_	1.81	0.405
Weekly internet use (min 0.5, max 42 hours, mean 23.49, SD 12.73); Mean (SE)	22.12 (0.19)_a_	27.11 (0.43)_b_	31.49 (0.81)_c_	168.06	0.001
Weekly social media use (min 0.5. max 42 hours, mean 23.13, SD 15.56); Mean (SE)	21.38 (0.23)_a_	27.68 (0.48)_b_	33.73 (0.83)_c_	264.26	0.001
Self-esteem (min 1, max 4, mean 2.73, SD 0.61); Mean (SE)	2.79 (0.01)_a_	2.54 (0.02)_b_	2.44 (0.04)_c_	155.10	0.001
Level of depressive symptoms (min 1, max 4, mean 1.93, SD 0.60); Mean (SE)	1.85 (0.01)_a_	2.163 (0.02)_b_	2.36 (0.05)_c_	210.12	0.001

*Note*: Different subscript letters (a, b, c) in the same row reflect significant (*p*< 0.05) difference between the means while same subscript letters in one row reflect non-significant difference between the means according to pair wised Wald χ^2^ test of mean equality for latent class predictors in mixture modeling (www.statmodel.com/download/meantest2.pdf).

### Suggesting a cutoff score for classification: Sensitivity and specificity analysis

Because of the lack of a clinically diagnosed group of problematic social media users, the third LPA class (i.e., those at-risk of problematic social media use) was used as the ‘gold standard’ to determine the optimal cut-off threshold to classify those at-risk of problematic use. Sensitivity, specificity, positive predictive value (PPV), negative predictive value (NPV), and accuracy of the BSMAS at all possible cut-off points were calculated (Table 3). Based on this analysis, a cut-off score of 19 points was suggested as the ideal threshold at and above which individuals are classified as at-risk of problematic social media use.

**Table 3:** Cut-off points based on the third class (i.e., those at-risk of problematic social media use) derived from the Latent Profile Analysis.

Cut-off points	*True positive*	*True negative*	*False positive*	*False negative*	*Sensitivity (%)*	*Specificity (%)*	*PPV (%)*	*NPV (%)*	*Accuracy (%)*
12	243	4304	1224	0	100	78	17	100	79
13	243	4635	895	0	100	84	21	100	84
14	243	4823	701	0	100	87	26	100	88
15	243	4986	539	0	100	90	31	100	91
16	240	5141	386	3	99	93	38	100	93
17	232	5249	278	9	96	95	45	100	95
18	219	5340	188	23	90	97	54	100	96
**19**	**199**	**5458**	**74**	**40**	**83**	**99**	**73**	**99**	**98**
20	177	5503	29	64	73	99	86	99	98
21	156	5517	17	85	65	100	90	98	98
22	126	5527	8	114	53	100	94	98	98
23	107	5530	4	133	45	100	96	98	98

*Note*: Bold data indicate that the cut-off score of 19 (and above) was selected as a result of the sensitivity and specificity analysis. PPV = positive predictive value; NPV = negative predictive value

In this case, the specificity is 99% and the sensitivity is 83% (i.e., only 1% of the non-problematic social media users are identified incorrectly as being at-risk of problematic use by the scale, while 17% of true cases of problematic social media users are missed). At this value, PPV is 73% and the NPV is 99%. In other words, 27% of the individuals with a positive test result are identified incorrectly, while only 1% of individuals with negative test result are identified incorrectly. This yields an accuracy of 98%. Increasing the cut-off point would result in more false negative cases, while decreasing it would increase the number of social media users labeled incorrectly (as being at-risk) by the screening instrument.

## Discussion

To assess the prevalence of problematic social media use in a reliable and a valid way, the psychometric properties of the BSMAS were tested. According to the results, the BSMAS demonstrated adequate psychometric properties regarding its factor structure, reliability, and validity.

Using a latent profile analysis on the six items of the Bergen Social Media Addiction Scale (BSMAS), the adolescent social media users were divided into three different classes, and the analysis demonstrated that 4.5% of participants could be classified as being at-risk. Previous studies have shown a wide range of prevalence rates due to various methodological issues such as convenience sampling, targeting mainly college students, and/ or having small sample sizes [15, 12, 26, 42, 43]. For instance, the prevalence of problematic social media users among Nigerian University undergraduates was 1.6% [37], whereas among Malaysian college students the reported prevalence was 47% [43]. The results of the present study belong to the more conservative prevalence estimations. Moreover, they correspond to the prevalence rates of the general problematic and/or addictive Internet use, that range between 1% [59] and 18.7% [60] according to the recent review [61].

Regarding validity of the BSMAS, the at-risk group showed the lowest self-esteem and the highest level of depressive symptoms and the most time spent on internet and social media use, and was therefore in line with previous research findings [22, 24].

In addition, adolescents that were at-risk of social media use were mainly female, and reported the greatest amount of internet and social media usage. Previous studies have found similar gender differences in problematic social media use [4, 6] and problematic internet use [61]. For instance, Rehbein and Mößle [62] found that among adolescents with problematic internet use, girls indicated that social media contributed most to their addiction, while boys also cited online pornography as a primary source of their problems.

Furthermore, the results of the present study showed that within the at-risk group the withdrawal component had the highest score. Therefore, withdrawal symptoms should be highlighted when developing prevention and treatment programs in school environments for adolescents being at-risk of problematic social media use. A cut-off point was calculated using the third LPA class as the “gold standard” to categorize the risky or problematic social media users among adolescents in the sample. The suggested cut-off value with the most adequate sensitivity and specificity values was 19 points. Although, the calculated score cannot replace a clinically validated cut-off point, it may be more beneficial than using completely ad-hoc thresholds [63].

Despite the study’s strengths (most notably the large nationally representative sample using psychometrically validated instruments), the study is not without its limitations. The study only included Hungarian adolescents as participants. Therefore, to further test the psychometric properties of the BSMAS, cross-cultural studies should also be conducted in the future using different adolescent groups in different countries and cultures. Moreover, the data were all self-report and self-report measures may lead to different response biases [64], such as social desirability bias (e.g., reporting more favorable behavior than the truth), memory recall bias (difficulty in remembering past events), and response style bias (e.g., scores may show central tendency or extreme response style).

It is also important to highlight that psychometric screening tools tend to overestimate the prevalence rates of disorders when the true prevalence rate of the disorder is low. For instance, an instrument with moderate sensitivity and specificity (i.e., 80.5% and 82.4%, respectively) at a 2.1% prevalence level of the problematic behavior has a positive predictive value of 8.9%. This means that out of 100 respondents who score positive on the test only about 9 are true clinical cases [65]. Consequently, survey-based prevalence rates should be interpreted cautiously to avoid overpathologizing every day behaviors [66]. More generally, the issue of addiction to social networking and social media is a controversial issue and many papers have questioned whether the activity can be considered an addiction at all [4, 27, 66–68].

The study had a cross-sectional design, therefore causality cannot be established regarding the risk factors. Future research should apply longitudinal designs to identify the contributing factors of the problematic behavior among social media users. It should also be noted that when completing the BSMAS, the participants may have had a different conception of social media use than intended by the developers of the BSMAS. For instance, on sites such as *Facebook*, many different activities can be carried out such as social networking, gaming and gambling. Although the BSMAS is only concerned with social networking, there is always the possibility that participants’ conception of social media use included some or all of these other activities and therefore problematic use might be including non-social networking activities.

## Conclusion

In conclusion, the results of the present study suggest that the Bergen Social Media Addiction Scale [11] is a psychometrically valid scale that is an appropriate tool to identify the signs of risky social media use among adolescents. This instrument may be especially useful in school environments to identify those adolescents who are at-risk of problematic social media use and therefore could be utilized in prevention and intervention programs (i.e., content-control software, counseling, cognitive-behavioral therapy; [69]).
